# Supplementary material for: PYL Family Genes from Liriodendron chinense Positively Respond to Multiple Stresses
Source: Plants (Basel). 2023 Jul 11;12(14):2609. doi: 10.3390/plants12142609 (PMC10386353; doi:10.3390/plants12142609)
Supplement: Supplementary file 1 [file plants-12-02609-s001.zip › Supplementary Tables S1 and S2.pdf]

Supplementary information

Supplementary Tables：

Supplementary Table S1. Specific primers of genes for quantitative real-time PCR (qPCR) analysis

Accession number	Primer name	Sequence (5'-3')
Lchi00864	Lchi00864-qPCR F	CGAGCATCCTGAAAGGTATAAGCA
	Lchi00864-qPCR R	GTCACGTCTCTTATACTCCCTACACC
Lchi01385	Lchi01385-qPCR F	TGGATAAAATGCCCCATATAACCC
	Lchi01385-qPCR R	CGTGTTGAAATGACCAGAATACCC
Lchi11622	Lchi11622-qPCR F	TGGCAAACTACCAATCCACGAC
	Lchi11622-qPCR R	TGTGAAGATGACCGTATCCTCC
Lchi13641	Lchi13641-qPCR F	AAACAAATCCTCTGATCAACTCCG
	Lchi13641-qPCR R	TGTCCGTTCATTGCCAAATCACC
Lchi16997	Lchi16997-qPCR F	TTCGATCAGCCACAGAAATATAAGCC
	Lchi16997-qPCR R	GACTTCACATTCACCTCCCGAAC
18S RNA	18S RNA-qPCR F	ATTTCTGCCCTATCAACTTTCG
	18S RNA-qPCR R	TTGTTATTTATTGTCACTACCTCCC

Supplementary Table S2. Specific primers designed for gene clone

Accession number	Primer name	Sequence (5'-3')
Lchi01385	Lchi01385-clone F	ATGGAAGAAGGAGAGAAATCAACG
	Lchi01385-clone R	CTCTCCCTTTTGATCCTCCTTC
Lchi11622	Lchi11622-clone F	ATGGTGTTCTACCAACAAACCT
	Lchi11622-clone R	TCAATAGCTGAGAGAATCTGACTC
Lchi16997	Lchi16997-clone F	ATGGTAGGAGAAGAGAGGTGGTG
	Lchi16997-clone R	TCAGTATCGGTCGATGGGCTC
